# Evidence for separate translocation pathways in determining cadmium accumulation in grain and aerial plant parts in rice

**DOI:** 10.1186/1471-2229-9-8

**Published:** 2009-01-21

**Authors:** Takayuki Kashiwagi, Kumiko Shindoh, Naoki Hirotsu, Ken Ishimaru

**Affiliations:** 1 National Institute of Agrobiological Sciences, Kannondai 2-1-2, Tsukuba, Ibaraki 305-8602, Japan; 2 National Food Research Institute, Kannondai 2-1-12, Tsukuba, Ibaraki 305-8642, Japan

## Abstract

**Background:**

Cadmium (Cd) translocation and accumulation in the grain and aerial plant parts of rice (*Oryza sativa *L.) is an important aspect of food safety and phytoextraction in areas with contaminated soil. Because control of Cd translocation and accumulation is likely to be determined by the plants genetics, the Cd contents of grain and the aerial parts of rice may be manipulated to improve food safety and for phytoextraction ability. This study studied Cd translocation and accumulation and their genetic control in aerial parts of rice to provide a starting point for improving food safety and phytoextraction in Cd-contaminated soils.

**Results:**

In the *japonica *rice cultivar "Nipponbare", Cd accumulated in leaves and culms until heading, and in culms and ears after heading. Two quantitative trait loci (QTLs) from *indica *cv. "Kasalath", *qcd4-1 *and *qcd4-2*, affect Cd concentrations in upper plant parts just before heading. Three near-isogenic lines (NILs) with *qcd4-1 *and *qcd4-2 *were selected from the "Nipponbare" background, and were analyzed for the effects of each QTL, and for interactions between the two QTLs. From the results compared between "Nipponbare" and each NIL, neither QTL influenced total Cd accumulation in aerial parts at 5 days after heading, but the interaction between two QTLs increased Cd accumulation. At 35 days after heading, *qcd4-2 *had increased Cd accumulation in the aerial plant parts and decreased translocation from leaves other than flag leaf, but interaction between the two QTLs increased translocation from leaves. NIL*qcd4-1,2 *accumulated higher concentrations of Cd in brown rice than "Nipponbare".

**Conclusion:**

Three types of Cd translocation and accumulation patterns demonstrated by NILs suggested that the accumulation of Cd in leaves and culms before heading, and translocation from them after heading are responsible for Cd accumulation in grain. Cd translocation from roots to culms and ears after heading may direct Cd to the aerial organs without influencing brown rice accumulation.

## Background

Cadmium (Cd) is a harmful heavy metal that may be released into the biosphere by modern industry [1-3]. On soil with low Cd concentration, plants grow normally, but accumulate Cd in their edible parts [4]. The high accumulation of Cd by consumption of food represents a potential human health hazard and international trade standards have been discussed to limit the levels of Cd in exported cereal grain [5,6]. Thus, there is a need to understand the physiological processes that control acquisition of Cd from soil solution by roots and mobilization of Cd in plants.

Phytoextraction of Cd from polluted soil using hyperaccumulator plants with high Cd tolerance (e.g. *Thlaspi caerulescens*) is one of the major engineering-based methods for environmental restoration. However, the use of hyperaccumulator plants might be not suitable for phytoextraction on soil with low Cd contamination levels, because of low Cd uptake efficiency relative to biomass, growth rate, and the cultivation system [7]. Recently, several phytoextraction crops which could be grown more efficiently with the local cultivation system were evaluated in soils with low levels of Cd contamination [8]. This study suggested the use of crops with high Cd accumulation levels might be effective for phytoextraction from soils with low Cd concentrations.

Cd accumulation in crops is determined by varietal differences as well as by environmental factors. Varietal differences in Cd accumulation were reported in several crops, including wheat (*Triticum sestivum *L.) [9,10], barley (*Hordeum vulgare *L.) [11], maize (*Zea mays *L.) [12,13], potato (*Solanum tuberosum *L.) [14], and rice (*Oryza sativa *L.) [15,16]. Yu et al. [17] reported that Cd concentrations in grains of 43 rice cultivars ranged from 0 to 0.37 mg kg^-1 ^under low Cd exposure (1.75 mg kg^-1 ^soil), and ranged from 0.30 to 2.19 mg kg^-1 ^under high Cd exposure (76.95 mg kg^-1 ^soil). Additionally, the translocation of absorbed Cd from roots to shoots differs greatly among rice cultivars [18]. It thus may be possible to breed new rice cultivars that accumulate low concentrations of Cd in grain but with greater simultaneous accumulation of Cd in the aerial parts for phytoextraction and soil remediation.

The development of a detailed rice molecular genetic map and DNA markers has made it easy to analyze quantitative trait loci (QTLs) for complex traits [19], and the use of near-isogenic lines (NILs) is an effective method for characterizing QTLs in detail [20]. NILs have been used to characterize a number of QTLs for agronomic traits in rice, including heading date [21,22], lodging resistance [23,24], and yield traits [25,26]. Rice QTLs have also been used to study phosphorus uptake under deficiency conditions. The results using NILs suggested that a QTL on chromosome 12 (*Pup1*) affects root growth and efficiency [27-29].

Genetic analysis of Cd accumulation has been reported in cereals. Penner et al. [30] identified two random amplified polymorphic DNA (RAPD) markers linked to a gene responsible for cadmium uptake in durum wheat. QTLs that putatively determine Cd accumulation in brown rice have been mapped on chromosomes 3, 6, and 8 [31]. However, these rice QTLs show different effects under upland and flooded conditions, and the mechanism of their control over Cd accumulation is not clear.

Cd absorbed by roots is transported *via *both xylem and phloem in plants [4]. Cd translocation from roots to shoots is driven by transpiration in leaves [32]. In durum wheat, it is suggested that Cd accumulation in grains may occur *via *phloem [33]. High Cd accumulation levels in durum wheat grain may be partially due to the elevated translocation of Cd from leaves and stems to maturing grain [34]. Additionally, Cd concentrations in grains correlated with Cd concentrations in shoots during the vegetative phase, and with translocation from roots to shoots in bread and durum wheats [35]. Cd accumulation in the edible parts is thus likely to be controlled by the general translocation properties of leaves, stems and roots *via *the xylem and phloem. Liu et al. [36] reported that Cd concentrations in rice grain positively correlate with Cd quantity accumulation in plant, Cd distribution ratios to aerial parts, and Cd distribution ratios from aerial parts to the grain. However, it is not yet clear how similar Cd translocation and accumulation are between rice and wheat.

Because control of Cd translocation and accumulation is likely to be partially controlled by the plants genetics, the Cd contents of grain and the aerial parts of rice may be manipulated to simultaneously improve food safety and Cd uptake for phytoextraction. In this study, Cd translocation and accumulation and their genetic control in aerial parts of rice were studied to provide a starting point for improving food safety and phytoextraction in Cd-contaminated soils.

## Results

### Accumulation and translocation of Cd in "Nipponbare"

**Figure 1:**
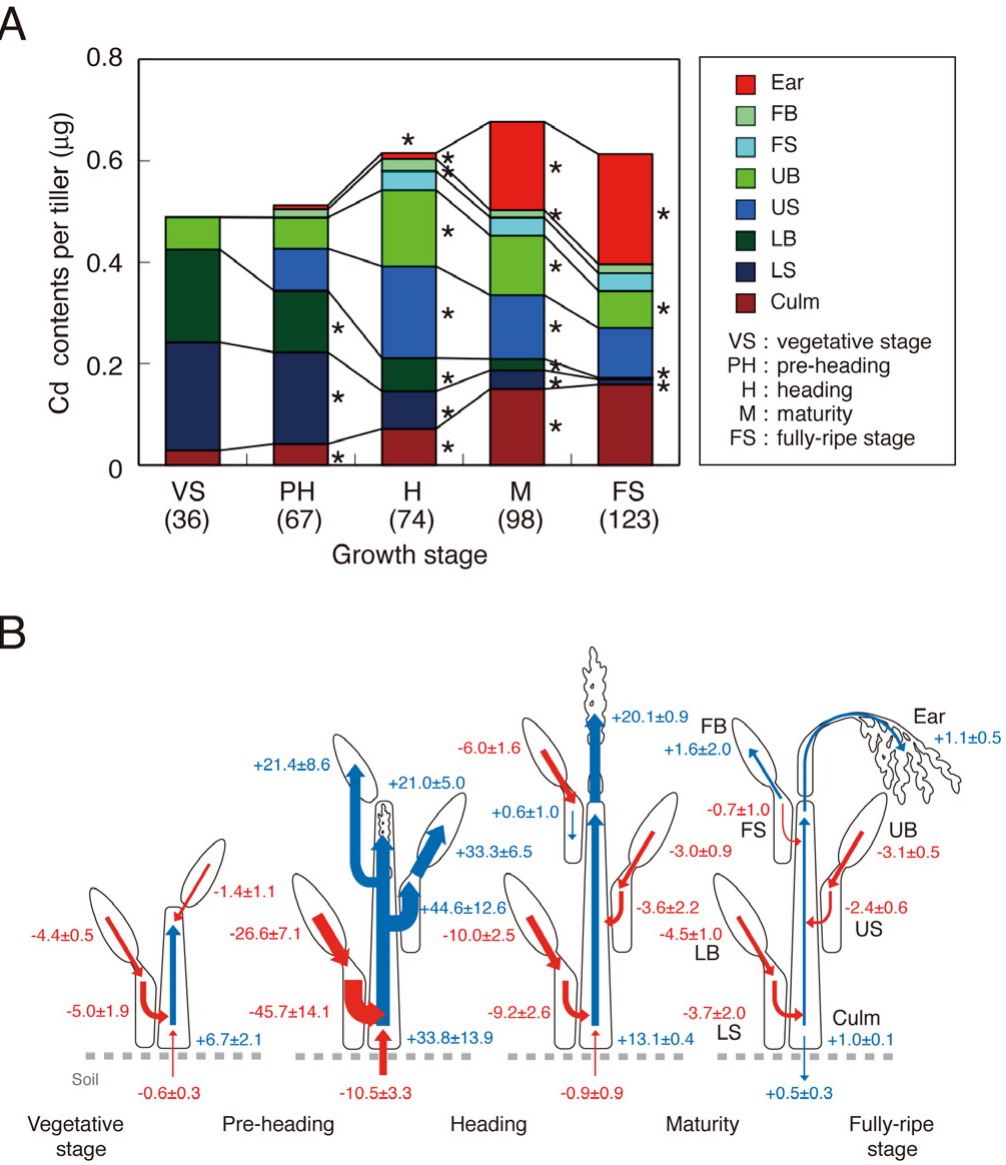
**Accumulation (A) and translocation (B) of Cd in "Nipponbare"**. Data represent a single tiller. Measurements were carried out at the vegetative, pre-heading, heading, maturity, and fully-ripe stages. Numbers in parentheses indicate days after transplanting (A). Asterisks on the right and the upper sides of bar indicate the significant differences of Cd contents in plant parts and the whole plant from the previous growth stage (*P *< 0.05), respectively. Arrows and values in blue indicate positive translocation rates (ng g dry wt^-1 ^d^-1^), and in red indicate negative translocation rates (B). Data are presented as means ± SE (n = 5).

Cd contents were determined in the ears (i.e. rachis, rachis branch, glumes, lemma, palea, and brown rice), flag leaf blades (FB), flag leaf sheaths (FS), upper leaf blades (UB), upper leaf sheaths (US), lower leaf blades (LB), lower leaf sheaths (LS), and culms of *Japonica *rice cultivar "Nipponbare" (Figure 1A). Among the growth stages, total Cd content per tiller of the whole plant was highest (0.655 ± 0.036 μg) at maturity (98 days after transplanting; DAT). Total content at pre-heading (67 DAT) was 75% of that at maturity (*P *< 0.01). Cd content increased in culms until the fully-ripe stage (123 DAT), which had 5.5 times the amount of Cd as the vegetative stage (*P *< 0.001). The Cd contents increase from heading to maturity was highest in culms (2.1 times, *P *< 0.001). The LB and LS accounted for 81% of the plant's total Cd at the vegetative stage (36 DAT), decreasing to 2% by the fully-ripe stage (*P *< 0.001). Cd content of the UB and US was highest at heading, accounting for 52% of the plant total contents. UB and US Cd contents decreased by the fully-ripe stage to 70% of that at maturity (*P *< 0.01). Cd accumulation in FB and FS was lower than in the other plant parts, decreasing only slightly after heading (*P *< 0.001). Cd contents increased in the ears after heading to 35% of that of the whole plant at the fully-ripe stage (*P *< 0.001).

Translocation rates of Cd were estimated between growth stages (Figure 1B; blue and red arrows indicate positive and negative translocation rates, respectively). LB showed the largest negative translocation from vegetation stage to pre-heading, compared with the other plant parts. From pre-heading to heading, Cd translocation from LB and LS, and uptake from paddy soil provided Cd to culms, UB, US, FB, and ears. Plant parts other than culms and FS, mainly LB and LS, provided Cd to culms and ears from heading to maturity. After maturity, Cd was translocated from leaves other than FB to culms, FB, and ears.

### QTL for Cd concentrations in upper plant parts just before heading

**Figure 2:**
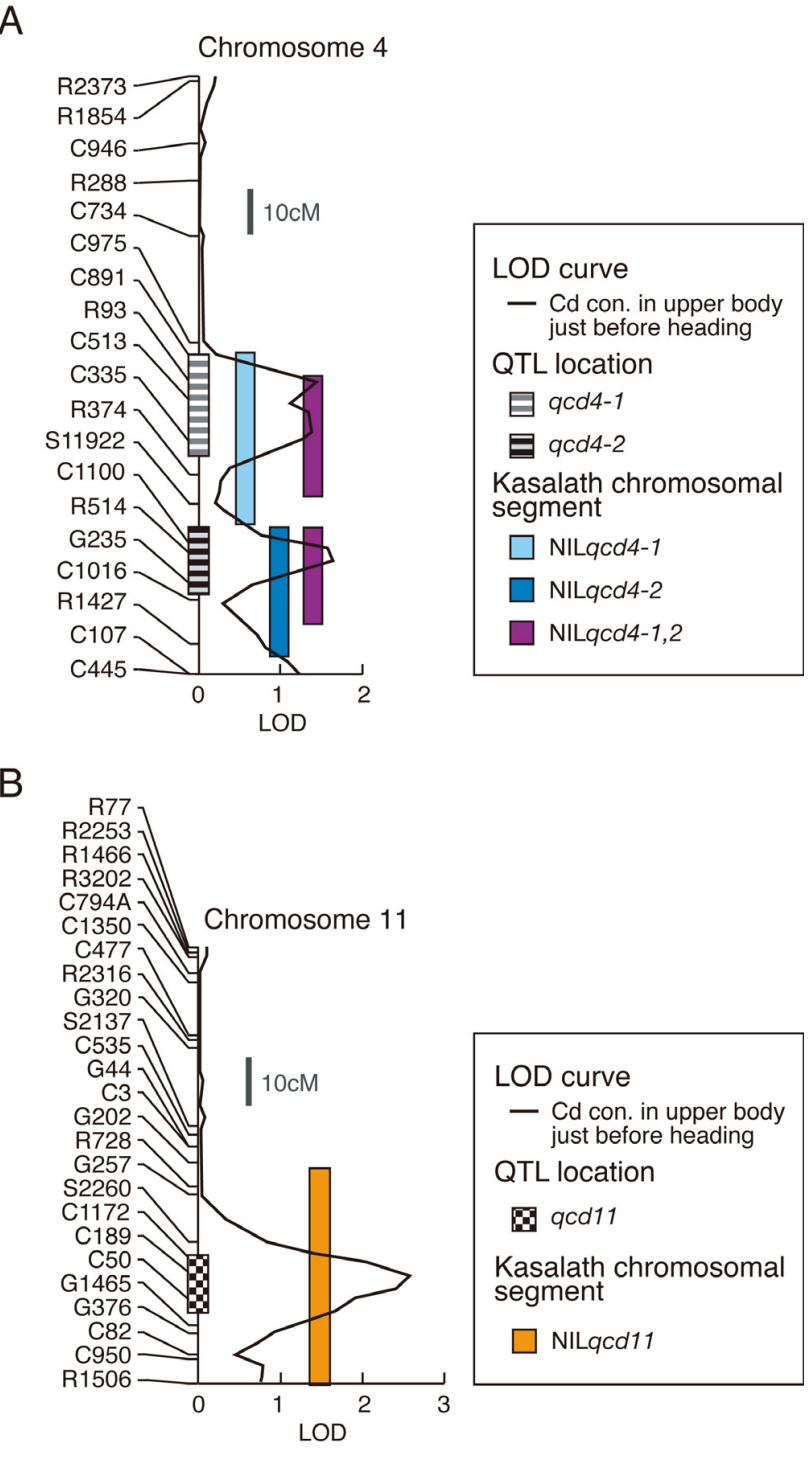
**LOD curves of QTLs and locations of "Kasalath" chromosomal segments in the NILs**. Two QTLs for Cd concentration in upper plant parts just before heading on chromosome 4 (A) and one QTL on chromosome 11 (B).

Cd concentrations in upper plant parts (i.e. from flag leaf to second leaf) just before heading were 0.341 ± 0.004 μg g^-1 ^dry wt in "Nipponbare" and 0.375 ± 0.015 μg g^-1 ^dry wt in "Kasalath", and were significantly different (*P *< 0.05). The mean Cd concentration in the upper plant parts of 98 BILs just before heading was 0.347 μg g^-1 ^dry wt, with a range of 0.258 ± 0.008 and 0.454 ± 0.004 μg g^-1 ^dry wt, respectively. QTLs for increasing and decreasing Cd concentrations in upper plant parts just before heading were detected on chromosomes 4 and 11, respectively (Table 1). Two QTLs on chromosome 4, tentatively named *qcd4-1 *and *qcd4-2*, had a positive effect with the "Kasalath" allele, and accounted for 0.082 and 0.079 (*r*^2^) of the phenotypic variations, respectively. *qcd4-1 *and *qcd4-2 *are located near the marker R93 and R514, and had 1.40 and 1.57 likelihood odds ratio (LOD) scores, respectively (Figure 2A and Table 1). A QTL on chromosome 11, tentatively named *qcd11*, had a negative effect with the "Kasalath" allele, and accounted for 0.105 (*r*^2^) of the phenotypic variations. It is located near the marker C189, and had an LOD score of 2.63 (Figure 2B and Table 1).

**Table 1:** QTLs for cadmium concentration in upper plant parts just before heading.

Chromosome no. (QTL name)	Marker locus nearest to putative QTL	LOD	DPE	*r*^2^
4 (*qcd4-1*)	R93	1.40	K	0.082
4 (*qcd4-2*)	R514	1.57	K	0.079
11 (*qcd11*)	C189	2.63	N	0.105

DPE, detection of phenotypic effect. N and K indicate whether "Nipponbare" or "Kasalath" alleles increased that value; *r*^2^, indicates phenotypic variation explained by each QTL.

### Selection of NILs with *qcd4-1*, *qcd4-2*, and *qcd11*

Among a series of chromosome segment substitution lines developed between "Nipponbare" and "Kasalath" by Yano's group at NIAS [20], we selected four NILs based on the unpublished genotyping data. NIL*qcd4-1 *contains a "Kasalath" chromosomal segment from C891 to S11922 underlying *qcd4-1 *in the "Nipponbare" genetic background (Figure 2A). NIL*qcd4-2 *contains a "Kasalath" chromosomal segment from C1100 to R1427 underlying *qcd4-2*. NIL*qcd4-1,2 *contains two "Kasalath" chromosomal segments, from R93 to R374 underlying *qcd4-1*, and from C1100 to C1016 underlying *qcd4-2*. NIL*qcd11 *contains a "Kasalath" chromosomal segment from R728 to R1506 underlying *qcd11 *(Figure 2B).

### Comparison of agronomic traits between "Nipponbare" and NILs

To analyze agronomic characters in NILs, we measured heading date, dry matter weight per tiller, and yield traits (Table 2). Each of the selected NILs headed from 1 to 4 days earlier than "Nipponbare". Only NIL*qcd4-1 *had lower tiller dry weights after heading and ear number than "Nipponbare", and there was no significant difference in grain number per plant between "Nipponbare" and NILs. Thousand-grain weights of the NILs were lower than in "Nipponbare", and only NIL*qcd4-1 *had significantly fewer ears per plant than "Nipponbare". NIL*qcd11 *headed one day later, and had higher grain number and lower 1,000-grain weights, than "Nipponbare" (data not shown). Tiller dry weights and ear number of NIL*qcd11 *were the same as of "Nipponbare".

**Table 2:** Comparison of agronomic traits between "Nipponbare" and NILs.

	Nipponbare	NIL*qcd4-1*	NIL*qcd4-2*	NIL*qcd4-1,2*
Days from transplanting to heading	76	72	73	75
Dry matter weight per tiller at 5 days after heading (g tiller^-1^)	3.21 ± 0.14	2.84 ± 0.13*	3.33 ± 1.11	3.45 ± 0.13
Dry matter weight per tiller at 35 days after heading (g tiller^-1^)	4.73 ± 0.12	4.33 ± 0.18*	4.88 ± 0.15	4.69 ± 0.10
**Yield traits**				
Number of ears per plant	12.8 ± 0.7	10.7 ± 0.7*	12.2 ± 0.9	14.1 ± 1.1
Number of grains per ear	84.6 ± 3.6	89.8 ± 2.7	90.7 ± 3.9	88.6 ± 2.6
1,000-grain weight (g)	21.5 ± 0.2	18.1 ± 0.1***	20.8 ± 0.1**	19.0 ± 0.1***

Data are expressed as means ± SE of nine plants; ***,**,*, Significant at the 0.001, 0.01 and 0.05 probability levels, respectively.

### QTL effects and influence on Cd concentrations in brown rice

Compared with "Nipponbare", three NILs containing *qcd4-1 *and *qcd4-2 *had significantly higher concentrations of Cd in their upper plant parts 5 days after heading (Table 3), indicating that the effects of *qcd4-1 *and *qcd4-2 *are consistently associated with differences in Cd concentrations. NIL*qcd11 *had significantly higher Cd concentrations in upper plant parts 5 days after heading than "Nipponbare" (1.2 times, data not shown). Because *qcd11 *has a negative effect with the "Kasalath" allele (Table 1), NIL*qcd11 *was excluded from further consideration in this study. By 35 days after heading, Cd concentrations in the upper plant parts of NIL*qcd4-1 *and NIL*qcd4-2 *were significantly higher than in "Nipponbare". Only NIL*qcd4-2 *increased Cd concentrations in its upper plant parts between 5 and 35 days after heading (1.1 times). The Cd concentration in "Nipponbare" brown rice (i.e. endosperm and embryo) was 0.018 ± 0.003 μg g^-1 ^dry wt, which was essentially the same as in NIL*qcd4-1 *and NIL*qcd4-2*, but the concentration of Cd in the brown rice of NIL*qcd4-1,2 *was 1.8 times as high as "Nipponbare" (Table 3).

**Table 3:** Cd concentrations in upper plant parts and brown rice of "Nipponbare" and NILs.

	Nipponbare	NIL*qcd4-1*	NIL*qcd4-2*	NIL*qcd4-1,2*
**Cd concentration in upper parts (μg g^-1 ^dry wt)**				
5 days after heading	0.260 ± 0.004	0.285 ± 0.006*	0.288 ± 0.006**	0.282 ± 0.004**
35 days after heading	0.263 ± 0.008	0.282 ± 0.006*	0.311 ± 0.010***	0.278 ± 0.010
**Cd concentration in grain (μg g^-1 ^dry wt)**				
Brown rice	0.018 ± 0.003	0.019 ± 0.002	0.019 ± 0.003	0.032 ± 0.004**

Data are expressed as means ± SE of six or more plants; ***,**,*, Significant at the 0.001, 0.01 and 0.05 probability levels, respectively

### NILs accumulation of Cd after heading

**Figure 3:**
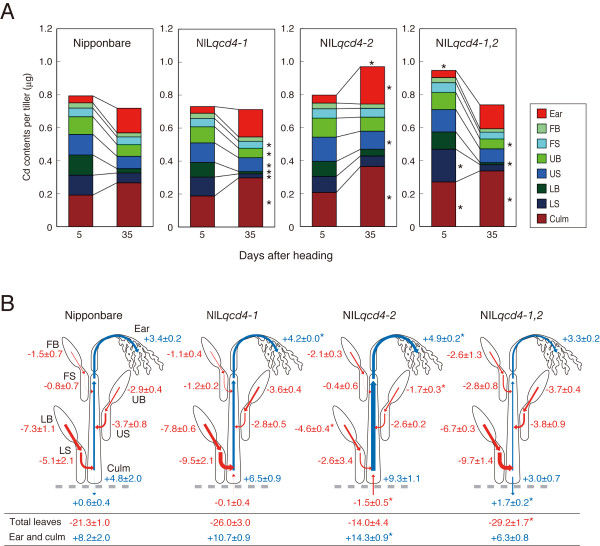
**Accumulation (A) and translocation (B) of Cd after heading in "Nipponbare" and NILs**. Data represent a single tiller from 5 to 35 days after heading. Numbers in parentheses indicate days after transplanting (A). Asterisks on the right and the upper sides of bar indicate the significant differences of Cd contents in plant parts and the whole plant compared with "Nipponbare" (*P *< 0.05), respectively. Arrows and values in blue indicate positive translocation rates (ng g dry wt^-1 ^d^-1^), and in red indicate negative translocation rates (B). Data are presented as means ± SE (n = 3). Asterisks indicate the significant differences compared with "Nipponbare" (*P *< 0.05).

By 5 days after heading, the Cd contents of "Nipponbare" culms was 0.92 ± 0.019 μg, and made up 24% of the total Cd contents of the whole plant (Figure 3A). Leaf blades and sheaths had 71% of the total Cd contents of whole "Nipponbare" plants. NIL*qcd4-1,2 *whole plant Cd content was significantly 20% higher than "Nipponbare" (*P *< 0.05). Culms and LS of NIL*qcd4-1,2 *were significantly 41 and 64% higher than "Nipponbare", respectively (*P *< 0.05).

At 35 days after heading, "Nipponbare" and NILs had increased Cd contents in culms and ears, with concomitant decreases in leaf blades and sheaths. NIL*qcd4-2 *total whole plant Cd content was significantly 35% higher than in "Nipponbare" (*P *< 0.05), but NIL*qcd4-1 *and NIL*qcd4-1,2 *were the same as "Nipponbare". NIL*qcd4-1 *and NIL*qcd4-1,2 *had significantly lower Cd content in LB (*P *< 0.05), and significantly higher contents in culms, compared with "Nipponbare" (*P *< 0.05). "Nipponbare" and NIL*qcd4-2 *had same total Cd contents in their leaves (leaf blades and sheaths, *P *> 0.05), but NIL*qcd4-1 *and NIL*qcd4-1,2 *had significantly lower total Cd contents in their leaves (*P *< 0.05). The Cd content of NIL*qcd4-2 *ears was 1.5 times as high as in "Nipponbare" (*P *< 0.01).

From 5 to 35 days after heading, the total Cd contents of NIL*qcd4-1,2 *significantly decreased by 22% (*P *< 0.01). Total Cd contents in parts other than the ears (leaf blades, sheaths, and culms) of NIL*qcd4-2 *did not change from 5 to 35 days after heading, but "Nipponbare" and the other NILs decreased (*P *< 0.05). The Cd contents of "Nipponbare", NIL*qcd4-1*, and NIL*qcd4-2 *leaves at 35 days after heading were 54, 49, and 70% as much as at 5 days, respectively (*P *< 0.05), and NIL*qcd4-1,2 *contents had decreased by 40% (*P *< 0.001). Among "Nipponbare" and the three NILs, NIL*qcd4-2 *had the highest increase in the rate of Cd accumulation in culms from 5 to 35 days after heading (1.8 times, *P *< 0.05), and NIL*qcd4-1,2 *had the lowest rate of accumulation (1.3 times, *P *< 0.01).

### Translocations of Cd after heading in NILs

In "Nipponbare", Cd translocations out of LB and LS from 5 to 35 days were -7.3 ± 1.0 and -5.1 ± 2.1 ng g dry wt^-1 ^d^-1^, which were the highest values of movement from source to sink (Figure 3B). Translocation rate out of total leaves in "Nipponbare" was -21.3 ± 1.0 ng g dry wt^-1 ^d^-1^. The primary sink organs were culms and ears (+4.8 ± 2.0 and +3.4 ± 0.2 ng g dry wt^-1 ^d^-1^, respectively). Translocation rates out of LB and UB in NIL*qcd4-2 *were significantly lower than in "Nipponbare" (*P *< 0.05). Translocation into the ears of NIL*qcd4-2 *was 45% higher than in "Nipponbare" (*P *< 0.01). Translocation rate out of total leaves in NIL*qcd4-1,2 *was significantly 37% higher than in "Nipponbare". Compared with "Nipponbare" and the other NILs, NIL*qcd4-2 *took up more Cd from paddy soil. In contrast, NIL*qcd4-1,2 *had higher outward translocation from aerial parts to paddy soil than "Nipponbare" (*P *< 0.05). On the sink side, the translocation rate of Cd into the ears of NIL*qcd4-1 *and NIL*qcd4-2 *were 1.2 and 1.5 times as high as of "Nipponbare", respectively (*P *< 0.05). The total translocation rate into the ears and the culm of NIL*qcd4-2 *was 1.7 times as high as of "Nipponbare" (*P *< 0.05).

## Discussion

Translocation and accumulation of Cd were assessed in rice cultivar "Niponbare" as part of an active strategy for modifying food safety and phytoextraction (Figure 1). Cd was translocated from lower leaves to upper leaves, and then from upper leaves to culms and ears. Lower leaves had the highest Cd contents during the early growth stages, but by the fully-ripe stage contained almost no Cd. These results suggest that the lower leaf tissues are primary Cd storage organs during the vegetative stage. From heading to fully-ripe, Cd contents decreased in upper and lower leaves and increased in culms and ears. The eventual Cd contents of the ears depend on translocation from both upper and lower leaf blades and sheaths, and on accumulation in the culm. Cd accumulation in wheat grain is the product of translocation from leaves and stems into maturing grain. Cd is accumulated in actively growing leaves and stems during vegetative growth, and is transported into the maturing seed head [34,35]. In rice, Cd contents in grain are determined by Cd already accumulated in leaves and stems before heading. Thus, Cd translocation from the vegetative to reproductive organs after heading is similar to what has been observed in bread and durum wheats.

Arao and Ae [15] reported that "Nipponbare" and "Kasalath" had similar Cd uptake efficiencies in their roots, but different translocation efficiencies to shoots and grain. Upper plant parts in cv. "Kasalath" had higher Cd concentrations than "Nipponbare" just before heading (data not shown). This study detected two QTLs (*qcd4-1 *and *qcd4-2*) that increase Cd concentrations in upper plant parts just before heading associated with the "Kasalath" allele (Figure 2A; Table 1). *qcd4-1 *and *qcd4-2 *might be a part of the genetic factors which determine differences in Cd translocation from root to shoot between "Nipponbare" and "Kasalath".

NIL*qcd4-2 *had translocated Cd from paddy soil to the aerial plant parts by 35 days after heading, and increased total Cd accumulation in the aerial plant parts (Figure 3A). *qcd4-2 *might be involved in Cd uptake in roots, either directly, or indirectly by affecting formation and functional maintenance of roots after heading. *qcd4-2 *did not increase Cd uptake after heading in the presence of *qcd4-1*, but the interaction between these QTLs increased total Cd accumulation in the aerial parts at 5 days after heading by higher Cd accumulation in culms and LS rather than just the upper plant part (Figure 3A). These observations indicate that total Cd contents in the aerial parts are determined by two factors, Cd accumulation in all parts of the plant before heading and Cd uptake in roots after heading. In this study, Nipponbare and NIL*qcd4-1,2 *showed decrease in total Cd contents in the aerial parts and outward translocation to paddy soil after heading in (Figure 3). This outward translocation means the translocation to root or the leakage to paddy soil (containing dead and fallen leaves on ground surface). Cd may partly translocate from the aerial parts to root after heading in rice, like potassium retranslocation from shoot to root [37,38]. The determination of total Cd contents in the aerial parts also involves the leakage to root or paddy soil.

Cd translocation property greatly differed among *qcd4-1*, *qcd4-2 *and their interaction. From 5 to 35 days after heading, LB and LS were largest source of Cd, and ears and culms were the main sinks (Figure 3B). *qcd4-2 *decreased Cd translocation out of UB and LB (Figure 3B). The interaction between *qcd4-1 *and *qcd4-2 *made higher translocation from total leaves, compared with "Nipponbare". *qcd4-*2 would inhibit Cd translocation out of leaf blades, and the combination of *qcd4-1 *and *qcd4-2 *would activate Cd translocation from overall leaves. *qcd4-1 *and *qcd4-2 *increased Cd translocation to ears, and only *qcd4-2 *increased total translocation to ears and culms (Figure 3B). These results suggest that the two QTLs have different Cd translocations from leaves to ears and culms, and that *qcd4-2 *has a stronger influence on translocation to the sink organ.

The higher Cd contents of NIL*qcd4-1,2 *brown rice might be caused by the increases in whole plant accumulation before heading and translocation from total leaves after heading through the interaction between *qcd4-1 *and *qcd4-2*, much like in bread and durum wheat [34,35]. Neither *qcd4-1 *nor *qcd4-2 *increased accumulation in brown rice, but interaction of the two QTLs significantly increased Cd (Table 3). The agronomic measured traits of NIL*qcd4-1,2*, "Nipponbare" and the other NILs were about the same (Table 2), but NIL*qcd4-1,2 *had higher total Cd accumulation at 5 days after heading than "Nipponbare" or the other NILs (Figure 3A). In rice, it was reported that Cd grain concentration in rice grain was largely governed by the transport of Cd from shoot to grain [36]. Additionally, the high accumulation of Cd in durum wheat grain at maturity reflects redistribution of Cd *via *the phloem pathway [39]. Therefore, the interaction between *qcd4-1 *and *qcd4-2 *may affect phloem-mediated translocation from leaves to brown rice after heading. NIL*qcd4-2 *showed higher Cd accumulation and translocation into ears after heading than "Nipponbare", but had same Cd concentration in brown rice (Figure 3, Table 3). Ears consist of rachis, rachis branch, glumes, lemma, palea, and brown rice. These results suggested that *qcd4-2 *might increase Cd accumulation of ear components other than brown rice. QTLs that decrease Cd accumulation in the aerial organs have great potential in rice breeding for food safety, because Cd concentration in rice grain correlates with Cd accumulation in the aerial parts [36]. In this study, *qcd11*, which should decrease Cd accumulation in the aerial plant parts before heading due to the "Kasalath" allele, was not found to be effective. Ishikawa et al. [31] detected two QTL for low Cd concentration in brown rice on chromosome 3 and 8, these QTL might decrease Cd accumulation in the aerial organs.

**Figure 4:**
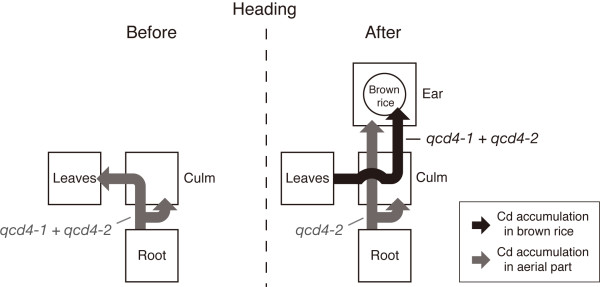
**Patterns of Cd translocation and accumulation in plant parts before and after heading**. Black and gray lines indicate Cd translocation associated with Cd accumulation in brown rice and aerial plant parts, respectively.

Cdtranslocation and accumulation before and after heading were affected by the QTLs in both an organ- and developmental stage-specific manner (Figure 4). From the vegetative stage to heading, Cd translocates from roots to aerial parts and accumulates in culms and leaves, and that translocation is increased by the interaction between *qcd4-1 *and *qcd4-2*. This pathway determines Cd content in aerial parts before heading. Cd accumulates in leaves until heading, and then it translocates to the ears *via *the culms, accumulating partly in brown rice. In this scenario, the interaction between *qcd4-1 *and *qcd4-2 *increases translocation from leaves and thus accumulation in brown rice (Figure 3, Table 3). It was suggested that Cd accumulation in grain might be responsible for phloem-mediated Cd translocation [33,39], therefore, this pathway might relate to Cd translocation to brown rice *via *phloem. An additional translocation and accumulation pathway translocates Cd from roots to culms and ears after heading. *qcd4-2 *apparently increases Cd translocation from roots to culms and ears through the second pathway after heading, resulting in greater Cd accumulation in aerial parts after heading (Figure 3). In ears, then, Cd accumulation increases at the components other than brown rice, e.g. rachis, glumes, etc (Figure 3, Table 3). This second pathway could affect Cd translocation from roots to shoots *via *xylem after hading without Cd translocation to brown rice. Harris and Taylor [40] suggested that restricted root-to-shoot Cd translocation might limit Cd accumulation in durum wheat grains by directly controlling Cd translocation from root during grain filling, or by controlling shoot Cd pools that can be remobilized to the grain. The second pathway after heading may limit Cd accumulation in brown rice by inhibiting Cd remobilization. Based on these patterns, the Cd translocation after heading takes two pathways, from leaves to brown rice and from roots to aerial parts. This study suggests that the differences in Cd translocation and accumulation before and after heading in rice are varietal, and that examining Cd flow in cultivars other than "Nipponbare" and "Kasalath" could clarify the whole picture of Cd translocation pathways.

## Conclusion

Cadmium accumulation in rice is affected by multiple cultivar-specific traits. The separate pathways of Cd translocation and accumulation before and after heading would be one of the determination factors of Cd contents in the grain or aerial plant parts. The data set used for this study has some limitations, namely use of NILs containing large Kasalath segments and phenotype analyses during a single year. For better clarification of the functions of *qcd4-1 *and *qcd4-2*, further studies are required to isolate a gene underlying QTL and to perform NIL experiments on different environments. In addition, Cd uptake of the plant is affected by soil Cd concentrations, thus further functional analyses of *qcd4-1 *and *qcd4-2 *under polluted soil condition will be required for improving the food safety of brown rice and the remediation efficiency of aerial plant part phytoextraction.

## Methods

### Plant materials

Rice (*Oryza sativa*) subsp. *japonica *cv. "Nipponbare" and subsp. *indica *cv. "Kasalath" accumulate low and medium concentrations of Cd, respectively, in brown rice [15]. "Nipponbare" was crossed with "Kasalath", and a F_1 _plant was backcrossed with "Nipponbare" to produce backcross inbred lines (BILs). Ninety-eight F_7 _BILs were developed from F_1 _BIL plants by the single-seed descent method [41]. The 98 F_7 _BILs and their parental lines were sown in mid-May 2000 and 2001. Seedlings were transplanted in early June with a single plant per hill, spaced at 18 × 30 cm and planted six plants of each line per row, and were grown on paddy field with alluvial soil (with approximately 0.24 mg Cd kg^-1^) under natural conditions (16.2–26.1°C of average daily temperature, published data by Japan Meteorological Agency [42]) ) in Tsukuba, Japan (latitude 36°N).

### QTL analysis

QTL analysis was performed by single-point analysis with the General Linear Model procedure of QGENE version 3.06 [43]. A probability level (*P*) of 0.01 was used as the threshold to detect significant differences in the mean values of two genotypic classes: homozygous for "Nipponbare" and homozygous for "Kasalath" alleles. To represent a QTL on a genetic map, we selected chromosome regions corresponding to a likelihood odds ratio (LOD) greater than the maxmum LOD minus 1, called an LOD-1 interval [44].

### Selection and growth conditions of near isogenic lines

From published genotyping data, we selected Near Isogenic Lines (NILs) from advanced backcross progeny of "Nipponbare" as the recurrent parent and "Kasalath" as the donor parent bred by Yano's group at the National Institute of Agrobiological Sciences [20]. NIL*qcd4-1*, NIL*qcd4-2*, and NIL*qcd4-1,2 *carry a chromosomal segment from "Kasalath" containing *qcd4-1*, *qcd4-2*, and both QTLs, respectively, on chromosome 4 in the "Nipponbare" genetic background (Figure 2A). NIL*qcd11 *carry a chromosomal segment from "Kasalath" containing *qcd11 *on chromosome 11 (Figure 2B). Four NILs and "Nipponbare" were sown in mid-May 2002, and were grown as above in a randomized design (different three spots on paddy field) to reduce the effects of environmental factors.

### Measurement of Cd rice plant tissues

To measure the translocation and accumulation of Cd plant organs were separated into ears (consisting of rachis, rachis branch, glumes, lemma, palea, and brown rice), flag leaf blades (FB), flag leaf sheaths (FS), first and second leaves below the flag leaf (upper leaf blades or sheaths, UB or US), leaf blades (LB) or sheaths (LS) below the second leaf, and culms. Tissues were sampled at the vegetative (36 days after transplanting; DAT), pre-heading (67 DAT), heading (74 DAT), maturity (98 DAT), and fully-ripe stages (123 DAT). To analyze QTLs for Cd concentration in the upper parts (i.e. from flag leaf to second leaf) of the plant body, BILs and their parental lines were sampled just before heading. The upper parts, ears, FB, FS, UB, US, LB, LS, and culms of three tillers per NILs plant were sampled 5 and 35 days after heading. Brown rice was sampled at the fully-ripe stage.

Cd concentrations in samples other than brown rice were measured by an energy-dispersive x-ray fluorescence spectrometer (EDXRF, element analyzer JSX-3201, JEOL, Tokyo). Samples were weighed after drying for 3 days at 80°C. Dried samples were powdered at 15,000 rpm for 90 s in a Wonder Blender (Osaka Chemical Co., Osaka). Powdered samples (0.2 g dry wt) were pressed at 15 kN cm^-2 ^in a hydraulic press (Evacuable KBr Die, Shimadzu, Kyoto) to form a 13-mm-diameter tablet. The x-ray intensity of Cd in the pellet was analyzed with EDXRF at 30 kV for 600 s and replicated three times for each sample, according to the method of Kashiwagi and Ishimaru [23]. Cd was analyzed as x-ray intensity at the peaks of 3.133, 3.316, and 3.528 keV. The determination of Cd concentrations in plant parts was carried out with the calibration curve between x-ray intensity and measurement value by inductively coupled plasma atomic emission spectrometry (ICP-AES). Cd concentrations in all parts of "Nipponbare" at the fully-ripe stage were measured with a sequential plasma emission spectrometer (ICPS-7000, Shimadzu, Kyoto) to form a calibration curve. 0.5 g of dried sample powder was mixed in 10 mL of 1 N HCl for 1 day. The sample solution was stabilized for 3 days, and was centrifuged at 13,000 rpm for 10 min, and the supernatant was filtered (0.45 μm pore size) to remove plant debris. Cd concentrations were determined at a peak of 228.802 nm by ICP-AES, and Cd contents were calculated as Cd concentration × dry weight of plant parts per tiller. Cd concentration in the upper plant parts was determined from six plants in each line, and Cd contents in other parts (ears, FB, FS, UB, US, LB, LS, and culms) from three plants in each line.

Cd concentrations in brown rice, which consists of endosperm and embryo, were determined by ICP-AES (JICP-PS3000 UV, Leeman Labs, New Hampshire), according to the method of Shindoh and Yasui [45] using nine plants in each line. Samples were dried at 135°C for 20 h, digested with HNO_3 _at 150°C, and re-digested with HClO_4 _at 210°C. The digested samples were dissolved in 1% HCl. The sample solutions were analyzed at a peak of 226.502 nm by ICP-AES.

Statistical differences between "Nipponbare" and NILs were determined using Student's *t*-test.

### Translocation rate of Cd in plant parts

Cd translocation rates (ng g dry wt^-1 ^d^-1^) between plant parts in different growth stages were calculated using the average rate of net ion uptake J_(R) _[46]. The calculation of translocation rate is as follows: J_(R) _= (M_2 _- M_1_)/(t_2 _- t_1_)·(ln(W_2_/W_1_))/(W_2 _- W_1_), where M is Cd content, M_2 _- M_1 _indicates the change in Cd content between growth stages, t_2 _- t_1 _is days between growth stages, and W is dry matter weight of the plant part. (ln(W_2_/W_1_))/(W_2 _- W_1_) is the average weight of the part [47]. Cd translocation rates in "Nipponbare" were calculated from the vegetative stage to pre-heading (31 days), from pre-heading to heading (7 days), from heading to maturity (24 days), and from maturity to the fully-ripe stage (25 days). To analyze QTLs effect, the translocation rates from 5 to 35 days after heading were calculated in "Nipponbare" and NILs. Statistical differences between "Nipponbare" and NILs were determined using Student's *t*-test.

### Measurement of Heading Date, Dry Matter Weights, and Yield Traits in NILs

To check other potential influences on the "Nipponbare" host due to chromosomal segments from "Kasalath", heading dates, dry matter weights, and yield traits were compared between "Nipponbare" and the NILs. At 5 and 35 days after heading, dried ears, FB, FS, UB, US, LB, LS, and culms were weighed, these sum were calculated as dry matter weight per tiller. The number of ears, grains per ear, and total weight of 1,000 rice grains (1,000-grain weight) were determined as yield traits from nine plants in each line. Statistical analysis of the differences between "Nipponbare" and the NILs was performed using Student's *t*-test.

## Authors' contributions

TK and KI designed the study and wrote most of the paper, and TK carried out most of the experimental works. KS carried out Cd measurement of brown rice. NH participated in QTL analysis and the phenotypic evaluation of NIL. All authors contributed to the manuscript preparation, and approved its final version.
